# G protein-coupled estrogen receptor expression in postnatal developing mouse retina

**DOI:** 10.3389/fopht.2024.1331298

**Published:** 2024-03-15

**Authors:** Wendy L. Piñon-Teal, Judith Mosinger Ogilvie

**Affiliations:** ^1^ Department of Biology, Saint Louis University, St. Louis, MO, United States; ^2^ Institute for Translational Neuroscience, Saint Louis University, St. Louis, MO, United States

**Keywords:** estrogen, estradiol (E2), G protein-coupled estrogen receptor 1 (GPER1), GPR30, retinal ganglion cells, neurodevelopment

## Abstract

**Introduction:**

Estrogen has emerged as a multifaceted signaling molecule in the retina, playing an important role in neural development and providing neuroprotection in adults. It interacts with two receptor types: classical estrogen receptors (ERs) alpha and beta, and G protein-coupled estrogen receptor (Gper). *Gper* differs from classical ERs in structure, localization, and signaling. Here we provide the first report of the temporal and spatial properties of *Gper* transcript and protein expression in the developing and mature mouse retina.

**Methods:**

We applied qRT-PCR to determine Gper transcript expression in wild type mouse retina from P0-P21. Immunohistochemistry and Western blot were used to determine *Gper* protein expression and localization at the same time points.

**Results:**

*Gper* expression showed a 6-fold increase during postnatal development, peaking at P14. Relative total *Gper* expression exhibited a significant decrease during retinal development, although variations emerged in the timing of changes among different forms of the protein. *Gper* immunoreactivity was seen in retinal ganglion cells (RGCs) throughout development and also in somas in the position of horizontal cells at early time points. Immunoreactivity was observed in the cytoplasm and Golgi at all time points, in the nucleus at early time points, and in RGC axons as the retina matured.

**Discussion:**

In conclusion, our study illuminates the spatial and temporal expression patterns of *Gper* in the developing mouse retina and provides a vital foundation for further investigations into the role of *Gper* in retinal development and degeneration.

## 1 Introduction

Estrogen is best known for its role in the development and regulation of the reproductive system. However, it also plays an important role in many organ systems, including the central nervous system (CNS) (1). Estrogen has been shown to have neuroprotective effects in the CNS (2) and in models of retinal degenerative disorders including glaucoma (3–5). Estrogen has also emerged as a multifaceted signaling molecule in CNS development, playing a role in cell proliferation, differentiation, migration, and growth of neuronal processes (1, 6–8).

Estradiol (E2), the predominant estrogen form, acts on two types of receptors, classical estrogen receptors (ERs) alpha and beta, and the non-canonical membrane estrogen receptor, G protein-coupled estrogen receptor (Gper). Gper is a novel estrogen receptor, differing from classical ERs in structure, localization, and signaling pathways (9, 10). Classical ERs are nuclear receptors that act through a genomic pathway, dimerizing when activated by E2 and translocating from the cytoplasm to the nucleus where they bind to promoters of target genes (11). In contrast, Gper regulates the rapid, non-genomic actions of E2 on classical ERs (9, 12–14), ultimately leading to genomic action (15). Gper, also known as Gper1 or GPR30, is highly conserved in vertebrate species (16, 17) dating to 4.5 million years ago. It is ubiquitously distributed throughout the body including the central and peripheral nervous systems (1). Gper regulates proliferation in neural stem/progenitor cells (18, 19) and differentiation in oligodendrocytes (20). In zebrafish, Gper has been linked to regulation of *Otx2* expression where it influences development of sensory organs and the brain (21). Here we provide the first report of the temporal and spatial properties of Gper transcript and protein expression in the postnatal developing and mature mouse retina.

## 2 Materials and methods

### 2.1 Animals

All animal procedures adhered to National Institutes of Health Guidelines on Laboratory Animal Welfare and were approved by Saint Louis University’s Institutional Animal Care and Use Committee (IACUC). Housing facilities were maintained with a 12-hour light/dark cycle. Tissue samples were harvested from wild type C57BL/6J mice (Jackson Laboratory, CAT #: 000664) at seven time points from postnatal day (P)0 to P21. Samples from animals were age-matched between different litters. Animals were sexed on P0 and again on the day of tissue collection. Animals between P0-P8 were euthanized via induced hypothermia followed by decapitation. Animals between P11-P21 were euthanized by intraperitoneal injection of pentobarbital (≤0.1 ml). Eyes were enucleated on ice and the anterior segment removed.

### 2.2 Quantitative reverse transcription polymerase chain reaction

Retinas were isolated as above and pooled with four retinas in each P0-P5 sample and two retinas in samples harvested at P8-P21. Total RNA was extracted using Quick RNA Mini-Prep Plus (Zymo Research, CAT #: R1057) using the manufacturer’s protocol. RNA quality was measured on the Synergy H1 BioTek plate reader at 260nm/280nm, and the values were between 1.8 and 2. cDNA was made with primers shown in **Table 1** using the Iscript Reverse Transcription Supermix (Bio-Rad, CAT#: 1708841) according to the manufacturer’s protocol on a Labnet MultiGene Optimax (Labnet International). The final concentration of cDNA for amplification was 903.12 ng (90.312 ng/μl). Samples were mixed with PowerUp SYBR Green Master Mix (Thermo Fisher, CAT #: A25742) and amplified in a MicroAmp Enduraplate Optical 96 well Fast Clear Reaction Plate (Applied Biosystems, CAT#: 4483485) covered with an Optical Adhesive Cover (Applied Biosystems, CAT#: 4360954) on a QuantStudio 5 Real-Time PCR System (Applied Biosystems).

**Table 1:** Primers for qRT-PCR.

Primer Name	Sequence	Size
Gper Forward	GCCTCTGCTACTCCCTCATC	20
Gper Reverse	ACTGCGAAGATCATCCTCAGG	21
Rplp0 Forward	ATCTGCTGCATCTGCTTG	18
Rplp0 Reverse	CGACCTGGAAGTCCAACTAC	20

### 2.3 Western blot assay

Tissue processing and Western blot assay were performed as previously described (22) with variations noted. Retinas were isolated, flash frozen, and stored in microcentrifuge tubes at -80°C until further processing. P0 samples consisted of eight retinas, P2-P8 samples consisted of six retinas, and P11-P21 samples consisted of four retinas. Whole brain cortex from P2 was used as a positive control. Sample lysis was performed with a mixture of Pierce Radio Immune Precipitation Assay (RIPA) buffer (Thermo Fisher Scientific, CAT #: 89900), 1% Mammalian Protease Inhibitor Cocktail (Sigma-Aldrich, CAT #: P8340) and 1% 0.1M of phenylmethylsulfonyl fluoride protease inhibitor (Sigma-Aldrich, CAT #: P-7626). Tissue was emulsified by sonification for 20 (P0-P14) or 40 seconds (P21, plus positive control) with the Sonic Dismembrator Model 100 (Thermo Fisher Scientific) on a setting of 1 and an output of 3 Watts.

Total protein of the samples was determined using a BCA assay (Pierce™ BCA Protein Assay Kit, CAT #: 23227) following manufacturer’s instructions and measured on the Synergy H1 BioTek plate reader. Sample protein concentration was normalized with RIPA buffer, except for the positive control, followed by addition of 5% beta-mercaptoethanol (Sigma-Aldrich, CAT #: M3148), and 35% LDS 4x TruPage LDS Buffer (Millipore, CAT #: PCg3009). Gel electrophoresis was performed on three biological replicates of each timepoint using mPage precast gels (EMD Millipore Corporation, CAT #: MP41G12) and mPage MOPS SDS Running Buffer (EMD Millipore Corporation, CAT #: MPMOPS) following manufacturer’s instructions. Precision Plus Protein Kaleidoscope (Bio-Rad, CAT #: 1610375) was used as our protein standard.

Proteins were transferred overnight at 25 V in mPage Transfer Buffer (EMD Millipore Corporation, CAT #: MPTRB). Blots were cut between the 50 kDa and 36 kDa protein markers and incubated concurrently with either a polyclonal rabbit GPR30 antibody targeted to the C-terminal for Gper (GeneTex, CAT #: GTX107748, 1:3000) or GAPDH rabbit monoclonal antibody (Cell Signaling Technologies, CAT #: 14C10,1:3000). The nitrocellulose was then incubated with IgG anti-rabbit horse radish peroxidase linked secondary antibody (Cell Signaling Technologies, CAT #: 7074S, 1:2500), with three subsequent washes for five minutes in PBST. Clarity Western ECL Substrate (Bio-Rad Laboratories, CAT #: 170-5060) was applied to the nitrocellulose as per the manufacturer’s protocol to visualize proteins via iBright FL1000.

### 2.4 Immunohistochemistry

Tissue processing and immunohistochemical procedures were as previously described (22). Briefly, eyecups were washed in 0.1M Phosphate Buffer Saline (PBS), fixed in 4% paraformaldehyde overnight, cryoprotected in a 30% sucrose solution, and embedded in OCT (Sakura, CAT#: 62550-01). The samples were immediately frozen on dry ice and stored at -80°C. Twelve-micron sagittal sections were cut on a cryostat (Leica CM 1850), adhered to gelatin-subbed slides, and stored at -80°C until stained. Anti-rabbit IgG Gper polyclonal antibody (GeneTex, CAT #: GTX107748, 1:1500) and anti-mouse IgG GM130 antibody (BD Biosciences, CAT #: 610822, 1:1500) were used to label Gper and the cis-Golgi marker, respectively. Secondary antibodies were Alexa Fluor 488 donkey anti-rabbit IgG (Molecular Probes, Eugene, OR; 1:600) and Alexa Fluor 555 donkey anti-mouse IgG (Life Technologies, CAT #: 1270147, 1:600). Slides were coverslipped with Invitrogen Prolong Gold Antifade Reagent with DAPI (Thermo Fisher Scientific, CAT #: P36932) and cured at room temperature for 24 hours prior to imaging on an SP8 Leica Confocal Microscope. Lasers, gain, zoom, format, a three-line average, and pinhole diameter were consistent for all images. Images were taken in 2048 x 2048 format. No adjustments were made to the images. Three or more retinal samples were analyzed at each timepoint. All images were taken from the central half of the retina.

### 2.5 Statistics and data analysis

qRT-PCR was performed with four biological replicates. Each plate included one biological sample and one technical replicate of that sample. Samples were separated by sex in addition to developmental timepoint and then normalized to the housekeeping gene *Rplp0*, which is reported to be highly stable across various tissues under different conditions, including developmental stages (23–25). Since no gender differences were observed in preliminary studies, data from males and females were combined for statistical analysis. The delta delta Ct method was used to calculate the relative fold change in *Gper* expression over time, followed by a one-way ANOVA to assess differences in gene expression fold changes.

Semiquantitative analysis of protein expression was determined using ImageJ densitometry to measure protein intensity on Western blot images. Integrated density measurements were recorded on three Gper bands and GAPDH. Integrated density values were averaged between four biological replicates for each timepoint and normalized to developmental timepoint P0. A one-way analysis of variance (ANOVA) was performed in Prism to analyze the effect of developmental time on Gper expression followed by a *post hoc* Tukey test for two-way comparisons between developmental timepoints. The 250 kDa Gper band exhibited insufficient density relative to the GAPDH control in early timepoints across two separate gels, precluding its reliable statistical assessment.

## 3 Results

### 3.1 *Gper* transcript expression in developing retina

**Figure 1:**
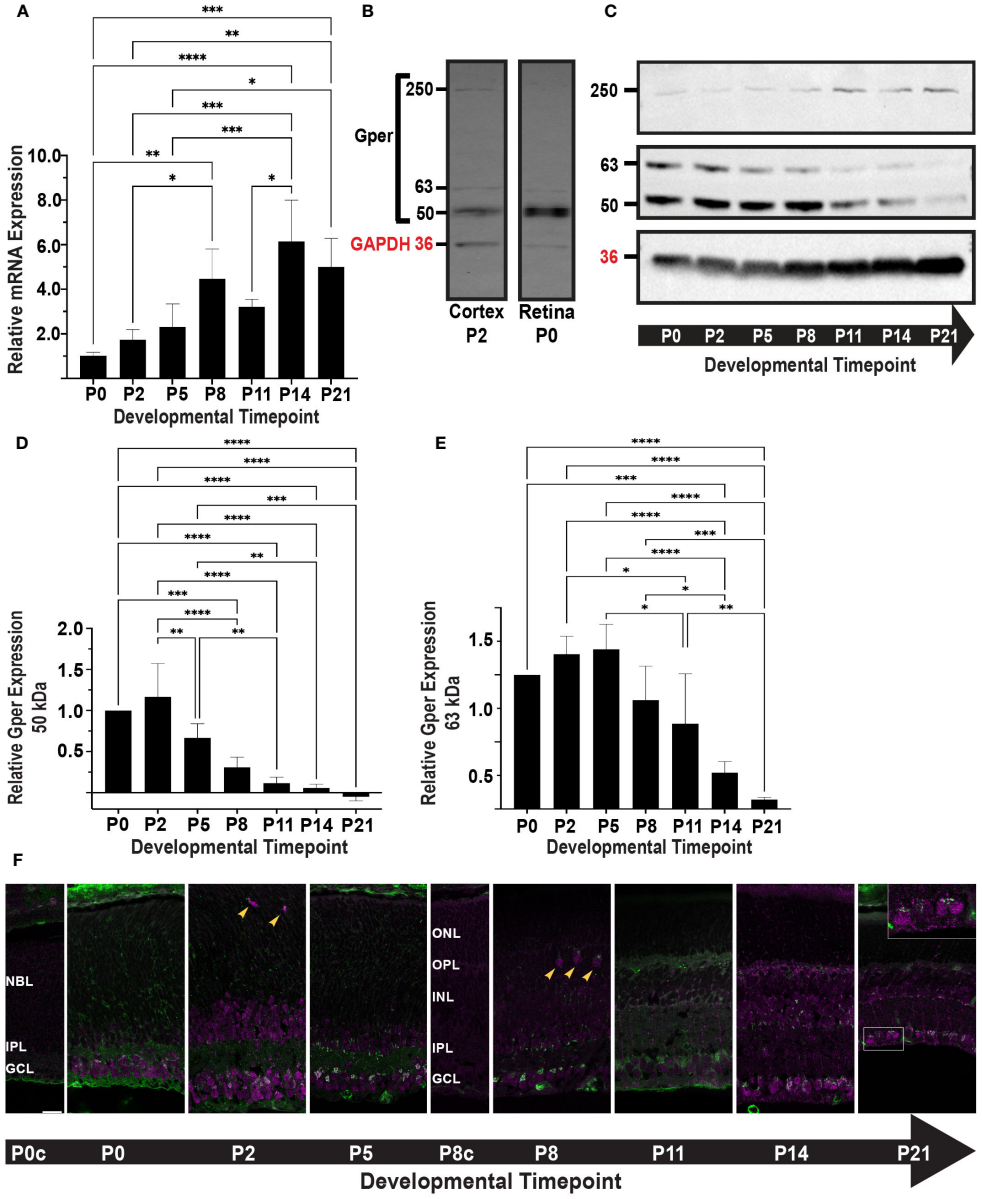
**(A)** *Gper* transcript expression was determined by qRT-PCR. The histogram displays the relative fold change in mRNA expression of *Gper* across seven timepoints, normalized to P0. Values represent the mean ± standard deviation from four biological replicates. *p<0.05, **p<0.01, ***p<0.001, ****p<0.0001. **(B)** Gper protein expression was determined by Western blot. P2 mouse brain cortex was used as a positive control for the Gper antibody. Gper bands at 50 kDa, 63 kDa, and 250 kDa were detected in both mouse cortex, as previously reported (26), and retina. A band representing GAPDH is shown at 36 kDa (red label). **(C)** Representative Western blot of Gper expression at seven timepoints is shown. GAPDH (36 kDa, red label) was used as a loading control. **(D)** The histogram displays the relative expression of 50 kDa Gper across seven timepoints, normalized to P0. Values represent the mean ± standard deviation from four independent experiments. **p<0.01, ***p<0.001, ****p<0.0001. **(E)** The histogram displays the relative expression of 63 kDa Gper across seven timepoints, normalized to P0. Values represent the mean ± standard deviation from four independent experiments. *p<0.05, **p<0.01, ***p<0.001, ****p<0.0001. **(F)** Gper (top) and GM130 (bottom) localization during postnatal retinal development. Mouse retinal sections were labeled with antibodies to Gper (magenta) and the cis-Golgi marker GM130 (green). Representative images from at least three samples at each of seven timepoints are shown. Punctate Gper staining and colocalization with GM130 (white) were most intense in the GCL. Gper immunoreactivity was observed in a small number of somas in the position of horizontal cells from P2-P8 (arrowheads). By P14, RGC somas and axons were more intensely labeled (P21 inset). Control sections from P0 (P0c) and P8 (P8c) retinas treated without primary antibody showed nonspecific GM130 staining of blood vessels and, at P8, nonspecific Gper staining in the developing outer segments. All images were taken from a single 12 μm slice and photographed at 63X with 0.75X zoom. Gper and GM130 staining are shown independently in the **Supplementary Figure**. NBL, neuroblast layer; ONL, outer nuclear layer; OPL, outer plexiform layer; INL, inner nuclear layer; IPL, inner plexiform layer; GCL, ganglion cell layer.

We determined the expression profile of *Gper* in the mouse retina during postnatal development, spanning seven distinct time points from P0 to P21. *Gper* expression showed a 6-fold increase during postnatal development, peaking at P14 (**Figure 1A**). Significant differences were confirmed by a one-way ANOVA test (p < 0.0001, F = 11.92, df = 6). Posthoc analysis indicated differences between early (P0-P5), mid (P5-P11), and late (P14-P21) timepoints with the greatest difference between P0 and P14 as illustrated in **Figure 1A**. No significant differences were observed within early, mid, and late developmental phases.

### 3.2 Gper protein expression in developing retina

Prior investigations have reported multiple specific bands produced by Gper antibodies in Western blot assays, including a 50 kDa band representing non-glycosylated Gper and one or more larger bands representing glycosylated Gper or detergent-resistant protein complexes (27, 28). P2 whole mouse brain cortex was used as a positive control (26) with an antibody that has previously been validated through shRNA and siRNA knockdown (29). Consistent with previous reports, distinct Gper bands were detected in P2 cortex and were further confirmed in P0 retina at 50 kDa, 63 kDa, and 250 kDa (**Figure 1B**).

We determined the expression profile of Gper protein in the mouse retina during postnatal development at seven time points from P0 to P21. The three bands identified in positive controls were apparent throughout development of the retina (**Figure 1C**). The 50 kDa band consistently exhibited greater intensity compared to the 63 kDa band, with both bands displaying a reduction over the course of development. The 50 kDa band decreased sharply beginning at P5 (**Figure 1D**), while the 63 kDa band peaked at P5 and decreased beginning around P8-P11 (**Figure 1E**). Observations of the 250 kDa glycosylated Gper were suggestive of an increase at later time points, which would follow the opposite trend to non-glycosylated Gper (**Figure 1C**), however the data was insufficient for quantitative analysis, precluding definitive conclusion.

### 3.3 Gper localization in developing retina

We next performed immunohistochemistry to determine the spatial and temporal expression of Gper during postnatal development. To better visualize changes in subcellular localization resulting from increased Gper glycosylation, retinal sections were double labeled with the cis-Golgi marker GM130 (**Figure 1F**; **Supplementary Figure**). Throughout development, Gper immunoreactivity had a speckled appearance within the ganglion cell layer (GCL) somas. Abundant staining was seen in both the cytoplasm and nucleus, with the highest intensity localized in the perinuclear region. Colocalization with GM130 confirmed that Gper perinuclear staining was in the Golgi apparatus.

Gper immunoreactivity was also seen in a population of cells in the inner portion of the neuroblast (NBL) and inner nuclear (INL) layers. A small number of somas in the position of horizontal cells displayed Gper immunoreactivity from P2 to P8 (**Figure 1F**, arrowheads). Overall, staining appeared diminished around P8, coinciding with a decrease in the 63 kDa Gper. By P11, Gper became distinctly discernible in individual processes, particularly those extending through the thickness of the inner plexiform layer (IPL), with the characteristic appearance of Müller glial cells. Staining in the Golgi and cytoplasm, but not the nucleus, became more prominent again at P14 and by P21, retinal ganglion cell (RGC) somas and axons were intensely labeled (**Figure 1F**, inset). Small, punctate Gper staining was seen in the outer nuclear layer (ONL) beginning at P14.

## 4 Discussion

Gper orchestrates rapid and transient responses through protein kinase A (PKA) signaling as well as transactivation of the epidermal growth factor receptor (EGFR) and Notch signaling pathways (15), in contrast to classical ERs that bind directly to promoters of target genes (11). It regulates the effects of E2 on classical ERs (9, 12–14) and is important for other cellular processes such as cell proliferation, migration, and ion channel regulation (30–34).

Our investigation reveals *Gper* expression throughout postnatal development in the mouse retina, peaking at P14. Notably, Gper immunoreactivity is evident in retinal ganglion cells (RGCs) from P0 to maturity, aligning with findings that *gper* is expressed in sensory regions of the fish CNS, including the retina (16, 21) and that *gper* expression is necessary for normal development of RGCs in zebrafish (8, 21). The continued expression of Gper in mature RGCs is noteworthy in light of the growing body of evidence indicating that estrogen plays a role in preserving RGC health and protecting against glaucoma (35). *Gper* is expressed in RGCs of adult mouse primary retinal cultures where it has neuroprotective effects against excitotoxicity and hypoxia (5, 36, 37). Estrogen, acting through the ERK pathway, has been shown to have a neuroprotective effect on axotomized RGCs, although it is unclear whether Gper or classical ERs are involved (38).

We observed a discrepancy between the qRT-PCR results, which indicated an increase in *Gper* mRNA expression throughout development (**Figure 1A**), and the Western blot results, which indicated a decline in Gper protein expression (**Figures 1D, E**). Divergence between mRNA and protein levels have been widely reported in the literature (39). Several biological or technical factors may contribute to this phenomenon, such as a decrease in the rate of translation, an increase in protein turnover due to changes in posttranslational modification, or the choice of housekeeping genes. Alternatively, post-translational modifications combined with the denaturation of protein required for electrophoresis may alter the protein’s conformation in a manner that masks it from antibody detection. This issue could be exacerbated for Gper due to glycosylation, which appears to increase during retinal development (**Figure 1C**). Using different tissues, antibodies, and experimental conditions, others have reported glycosylated Gper at molecular weights that we did not observe (27, 28), raising the possibility that a substantial amount of glycosylated protein remains present but undetected. This technical explanation most closely aligns with our immunohistochemical observations that exhibit robust Gper staining at later developmental timepoints, when Gper detection on Western blots was greatly diminished.

We observed transient Gper immunoreactivity in the inner portion of the INL in presumptive progenitor cells during a period of cell cycle exit, differentiation, and neurite outgrowth (40, 41). Studies in other tissues support a role for Gper in mediating these processes. During CNS development, estrogen receptors, including Gper, play a crucial role in regulating proliferation, differentiation, and neurite outgrowth in progenitor cells through modulation of genes that regulate cell cycle progression and cell fate determination (18, 34, 42). It contributes to proliferation and migration of cells in bone marrow mesenchymal stem cells and in a variety of cancers (43, 44). Gper regulates cyclin genes, which are important for both cell proliferation and differentiation (43), and acts via the PI3K/Akt pathway to modulate cadherins and other transcription factors that are necessary for cell migration and proper neurite outgrowth (45, 46).

Gper and estrogen signaling are known to regulate expression of the transcription factor *Otx2* in the CNS (21, 47). *Otx2* is essential for eye development and photoreceptor cell fate determination (48). Single-cell RNA-sequencing has identified early expression of *Gper* in the mouse retina, but expression levels are too low for accurate analysis (49). Since *Otx2* is activated embryonically in the mouse retina, future studies should investigate whether *Gper* is transiently expressed in precursor cells prior to *Otx2* expression. Punctate Gper immunostaining was observed in ONL beginning at P14, a time in which photoreceptors are extending their outer segments and Müller glial cells are extending processes through the ONL. Identification of the cell types labeled by Gper will require further studies with cell-specific markers.

Subcellular localization of Gper has been reported by some investigators on the cell membrane (50), as is the case for other GPCRs, but also intracellularly in the ER, Golgi, and nucleus by others (9, 51). Differences may be due to variations in cell type, experimental conditions, and metabolic status. These factors and others can alter protein glycosylation, which plays an important role in protein trafficking, cellular localization, and stabilization of proteins and protein complexes, as well as modulating neuronal activity in the CNS (52). Changes in glycosylation of Gper during postnatal retinal development is likely indicative of changes in subcellular localization and function. In our Western blot experiments, the 250 kDa glycosylated Gper band was too faint relative to the GAPDH loading control for statistical analysis. Pretreating samples with an endoglycosidase to remove glycans would provide a means to investigate this question in future experiments (28, 53).

Colocalization studies reveal Gper association with the Golgi marker GM130 and punctate cytoplasmic immunostaining indicative of ER and/or vesicular localization. Notably, the nuclear localization of Gper, with a speckled appearance, diminished over time corresponding with a decrease in non-glycosylated Gper. *Gper* has a nuclear localization sequence, and recent reports show Gper can be transported into the nucleus if it is not glycosylated at N-44 (14, 54). In these studies, cell migration was dependent on nuclear Gper (54). These observations align with the hypothesis that non-glycosylated nuclear Gper may coordinate cell migration of progenitor cells. We did not observe any clear indication of cell membrane localization, however, we cannot eliminate the possibility that some Gper was present on the cell surface.

In conclusion, our study illuminates the spatial and temporal expression patterns of Gper in the postnatal developing mouse retina and provides a vital foundation for further investigations. Future research should aim to elucidate the role of Gper in retinal cell proliferation, specification, and/or neurite outgrowth and to determine its mechanism of action during retinal development. An important aspect of these studies will be investigation of Gper during embryonic development when proliferation and specification of many retinal cell types occur. Additionally, exploring Gper’s function in mature RGCs holds significant promise in elucidating the role of estrogen signaling in retinal health and disease. The convergence of these insights promises to extend our understanding of retinal development and function with the potential to provide insights into novel approaches to mediation of glaucoma and other retinal diseases.

## Data availability statement

The raw data supporting the conclusions of this article will be made available by the authors, without undue reservation.

## Ethics statement

The animal study was approved by Saint Louis University Institutional Animal Care and Use Committee. The study was conducted in accordance with the local legislation and institutional requirements.

## Author contributions

WP: Conceptualization, Data curation, Formal analysis, Funding acquisition, Investigation, Methodology, Validation, Writing – original draft, Writing – review & editing. JO: Conceptualization, Data curation, Formal analysis, Funding acquisition, Methodology, Supervision, Validation, Writing – original draft, Writing – review & editing.

## Supplementary material

Supplementary Figure Gper (top) and GM130 (bottom) localization during postnatal retinal development. See legend for **Figure 1F** for details.

## References

[1] Bustamante-Barrientos FA Mendez-Ruette M Ortloff A Luz-Crawford P Rivera FJ Figueroa CD. The impact of estrogen and estrogen-like molecules in neurogenesis and neurodegeneration: beneficial or harmful? Front Cell Neurosci. (2021) 15:636176. doi: 10.3389/fncel.2021.636176 33762910 PMC7984366

[2] Wise PM Dubal DB Wilson ME Rau SW Bottner M. Minireview: neuroprotective effects of estrogen-new insights into mechanisms of action. Endocrinology. (2001) 142:969–73. doi: 10.1210/endo.142.3.8033 11181507

[3] Cascio C Guarneri R Russo D De Leo G Guarneri M Piccoli F. Pregnenolone sulfate, a naturally occurring excitotoxin involved in delayed retinal cell death. J Neurochem. (2000) 74:2380–91. doi: 10.1046/j.1471-4159.2000.0742380.x 10820199

[4] Cascio C Guarneri R Russo D De Leo G Guarneri M Piccoli F. A caspase-3-dependent pathway is predominantly activated by the excitotoxin pregnenolone sulfate and requires early and late cytochrome c release and cell-specific caspase-2 activation in the retinal cell death. J Neurochem. (2002) 83:1358–71. doi: 10.1046/j.1471-4159.2002.01229.x 12472890

[5] Jiang M Ma X Zhao Q Li Y Xing Y Deng Q. The neuroprotective effects of novel estrogen receptor GPER1 in mouse retinal ganglion cell degeneration. Exp Eye Res. (2019) 189:107826. doi: 10.1016/j.exer.2019.107826 31586450

[6] Li Y Wang JP Santen RJ Kim TH Park H Fan P. Estrogen stimulation of cell migration involves multiple signaling pathway interactions. Endocrinology. (2010) 151:5146–56. doi: 10.1210/en.2009-1506 PMC2954727 20861240

[7] Ferreira A Caceres A. Estrogen-enhanced neurite growth: evidence for a selective induction of Tau and stable microtubules. J Neurosci Off J Soc Neurosci. (1991) 11:392–400. doi: 10.1523/JNEUROSCI.11-02-00392.1991 PMC6575216 1899446

[8] Cohen A Popowitz J Delbridge-Perry M Rowe CJ Connaughton VP. The role of estrogen and thyroid hormones in zebrafish visual system function. Front Pharmacol. (2022) 13:837687. doi: 10.3389/fphar.2022.837687 35295340 PMC8918846

[9] Revankar CM Cimino DF Sklar LA Arterburn JB Prossnitz ER. A transmembrane intracellular estrogen receptor mediates rapid cell signaling. Science. (2005) 307:1625–30. doi: 10.1126/science.1106943 15705806

[10] Thomas P Pang Y Filardo EJ Dong J. Identity of an estrogen membrane receptor coupled to a G protein in human breast cancer cells. Endocrinology. (2005) 146:624–32. doi: 10.1210/en.2004-1064 15539556

[11] Prossnitz ER Barton M. Estrogen biology: new insights into GPER function and clinical opportunities. Mol Cell Endocrinol. (2014) 389:71–83. doi: 10.1016/j.mce.2014.02.002 24530924 PMC4040308

[12] Prossnitz ER Barton M. The G-protein-coupled estrogen receptor GPER in health and disease. Nat Rev Endocrinol. (2011) 7:715–26. doi: 10.1038/nrendo.2011.122 PMC3474542 21844907

[13] Prossnitz ER Hathaway HJ. What have we learned about GPER function in physiology and disease from knockout mice? J Steroid Biochem Mol Biol. (2015) 153:114–26. doi: 10.1016/j.jsbmb.2015.06.014 PMC4568147 26189910

[14] Pupo M Maggiolini M Musti AM. GPER mediates non-genomic effects of estrogen. Methods Mol Biol. (2016) 1366:471–88. doi: 10.1007/978-1-4939-3127-9_37 26585158

[15] Prossnitz ER Barton M. The G protein-coupled oestrogen receptor GPER in health and disease: an update. Nat Rev Endocrinol. (2023) 19:407–24. doi: 10.1038/s41574-023-00822-7 PMC10187525 37193881

[16] Mangiamele LA Gomez JR Curtis NJ Thompson RR. GPER/GPR30, a membrane estrogen receptor, is expressed in the brain and retina of a social fish (Carassius auratus) and colocalizes with isotocin. J Comp Neurol. (2017) 525:252–70. doi: 10.1002/cne.24056 PMC5138143 27283982

[17] Thomas P Alyea R Pang Y Peyton C Dong J Berg AH. Conserved estrogen binding and signaling functions of the G protein-coupled estrogen receptor 1 (GPER) in mammals and fish. Steroids. (2010) 75:595–602. doi: 10.1016/j.steroids.2009.11.005 19931550 PMC2885585

[18] Zhang L Ma Y Liu M Ma Y Guo H. The effects of various estrogen doses on the proliferation and differentiation of cultured neural stem cells. Gen Physiol Biophys. (2019) 38:417–25. doi: 10.4149/gpb_2019022 31411572

[19] Zhong J Ge HF Zhang C Chen JY Li HH Fang XY. G protein-coupled estrogen receptor 1 negatively regulates the proliferation of mouse-derived neural stem/progenitor cells via extracellular signal-regulated kinase pathway. Brain Res. (2019) 1714:158–65. doi: 10.1016/j.brainres.2019.02.024 30797747

[20] Okada M Makino A Nakajima M Okuyama S Furukawa S Furukawa Y. Estrogen stimulates proliferation and differentiation of neural stem/progenitor cells through different signal transduction pathways. Int J Mol Sci. (2010) 11:4114–23. doi: 10.3390/ijms11104114 PMC2996786 21152324

[21] Shi Y Liu X Zhu P Li J Sham KW Cheng SH. G-protein-coupled estrogen receptor 1 is involved in brain development during zebrafish (Danio rerio) embryogenesis. Biochem Biophys Res Commun. (2013) 435:21–7. doi: 10.1016/j.bbrc.2013.03.130 23583372

[22] Dickison VM Richmond AM Abu Irqeba A Martak JG Hoge SC Brooks MJ. A role for prenylated rab acceptor 1 in vertebrate photoreceptor development. BMC Neurosci. (2012) 13:152. doi: 10.1186/1471-2202-13-152 23241222 PMC3576285

[23] Huo Y Li X Sun C Pan Z Li Q Du X. From stability to reliability: Unveiling the un-biased reference genes in porcine ovarian granulosa cells under different conditions. Gene. (2023) 897:148089. doi: 10.1016/j.gene.2023.148089 38123003

[24] Luo N Zhou Y Chen X Zhao Y Hu Y. Screening the optimal housekeeping genes (HKGs) of placenta tissues by RNA-sequence and qRT-PCR throughout gestation in goat (Capra Hircus). Gene. (2024) 895:147966. doi: 10.1016/j.gene.2023.147966 37972698

[25] Ren J Zhang N Li X Sun X Song J. Identification of reference genes for gene expression studies among different developmental stages of murine hearts. BMC Dev Biol. (2021) 21:13. doi: 10.1186/s12861-021-00244-6 34496746 PMC8425138

[26] Hazell GG Yao ST Roper JA Prossnitz ER O’Carroll AM Lolait SJ. Localisation of GPR30, a novel G protein-coupled oestrogen receptor, suggests multiple functions in rodent brain and peripheral tissues. J Endocrinol. (2009) 202:223–36. doi: 10.1677/JOE-09-0066 PMC2710976 19420011

[27] Maiti K Paul JW Read M Chan EC Riley SC Nahar P. G-1-activated membrane estrogen receptors mediate increased contractility of the human myometrium. Endocrinology. (2011) 152:2448–55. doi: 10.1210/en.2010-0979 21427217

[28] Sanden C Broselid S Cornmark L Andersson K Daszkiewicz-Nilsson J Martensson UE. G protein-coupled estrogen receptor 1/G protein-coupled receptor 30 localizes in the plasma membrane and traffics intracellularly on cytokeratin intermediate filaments. Mol Pharmacol. (2011) 79:400–10. doi: 10.1124/mol.110.069500 21149639

[29] Tsai CL Wu HM Lin CY Lin YJ Chao A Wang TH. Estradiol and tamoxifen induce cell migration through GPR30 and activation of focal adhesion kinase (FAK) in endometrial cancers with low or without nuclear estrogen receptor alpha (ERalpha). PloS One. (2013) 8:e72999. doi: 10.1371/journal.pone.0072999 24039841 PMC3767783

[30] Evanson KW Goldsmith JA Ghosh P Delp MD. The G protein-coupled estrogen receptor agonist, G-1, attenuates BK channel activation in cerebral arterial smooth muscle cells. Pharmacol Res Perspect. (2018) 6:e00409. doi: 10.1002/prp2.409 29938113 PMC6011940

[31] Greenlee MM Mitzelfelt JD Yu L Yue Q Duke BJ Harrell CS. Estradiol activates epithelial sodium channels in rat alveolar cells through the G protein-coupled estrogen receptor. Am J Physiol Lung Cell Mol Physiol. (2013) 305:L878–89. doi: 10.1152/ajplung.00008.2013 PMC3882529 24097558

[32] Madeo A Maggiolini M. Nuclear alternate estrogen receptor GPR30 mediates 17beta-estradiol-induced gene expression and migration in breast cancer-associated fibroblasts. Cancer Res. (2010) 70:6036–46. doi: 10.1158/0008-5472.CAN-10-0408 20551055

[33] Sharma G Prossnitz ER. Mechanisms of estradiol-induced insulin secretion by the G protein-coupled estrogen receptor GPR30/GPER in pancreatic beta-cells. Endocrinology. (2011) 152:3030–9. doi: 10.1210/en.2011-0091 PMC3138237 21673097

[34] Haumann I Sturm MA Anstotz M Rune GM. GPER1 signaling initiates migration of female V-SVZ-derived cells. iScience. (2020) 23:101077. doi: 10.1016/j.isci.2020.101077 32361597 PMC7200306

[35] Fotesko K Thomsen BSV Kolko M Vohra R. Girl power in glaucoma: the role of estrogen in primary open angle glaucoma. Cell Mol Neurobiol. (2022) 42:41–57. doi: 10.1007/s10571-020-00965-5 33040237 PMC11441221

[36] Li R Wang Y Chen P Meng J Zhang H. G-protein-coupled estrogen receptor protects retinal ganglion cells via inhibiting endoplasmic reticulum stress under hyperoxia. J Cell Physiol. (2021) 236:3780–8. doi: 10.1002/jcp.30149 33151568

[37] Cao W Rajala RV Li F Anderson RE Wei N Soliman CE. Neuroprotective effect of estrogen upon retinal neurons in vitro. Adv Exp Med Biol. (2003) 533:395–402. doi: 10.1007/978-1-4615-0067-4_50 15180290

[38] Nakazawa T Takahashi H Shimura M. Estrogen has a neuroprotective effect on axotomized RGCs through ERK signal transduction pathway. Brain Res. (2006) 1093:141–9. doi: 10.1016/j.brainres.2006.03.084 16696958

[39] Greenbaum D Colangelo C Williams K Gerstein M. Comparing protein abundance and mRNA expression levels on a genomic scale. Genome Biol. (2003) 4:117. doi: 10.1186/gb-2003-4-9-117 12952525 PMC193646

[40] Heavner W Pevny L. Eye development and retinogenesis. Cold Spring Harb Perspect Biol. (2012) 4. doi: 10.1101/cshperspect.a008391 PMC3504437 23071378

[41] Reese BE. Development of the retina and optic pathway. Vision Res. (2011) 51:613–32. doi: 10.1016/j.visres.2010.07.010 PMC2974959 20647017

[42] Pemberton K Rosato M Dedert C DeLeon C Arnatt C Xu F. Differential effects of the G-protein-coupled estrogen receptor (GPER) on rat embryonic (E18) hippocampal and cortical neurons. eNeuro. (2022) 9. doi: 10.1523/ENEURO.0475-21.2022 PMC9291730 35788105

[43] Chuang SC Chen CH Chou YS Ho ML Chang JK. G Protein-Coupled Estrogen Receptor Mediates Cell Proliferation through the cAMP/PKA/CREB Pathway in Murine Bone Marrow Mesenchymal Stem Cells. Int J Mol Sci. (2020) 21:6490. doi: 10.3390/ijms21186490 32899453 PMC7555423

[44] Pepermans RA Sharma G Prossnitz ER. G protein-coupled estrogen receptor in cancer and stromal cells: functions and novel therapeutic perspectives. Cells. (2021) 10:672. doi: 10.3390/cells10030672 33802978 PMC8002620

[45] Pupo M Pisano A Abonante S Maggiolini M Musti AM. GPER activates Notch signaling in breast cancer cells and cancer-associated fibroblasts (CAFs). Int J Biochem Cell Biol. (2014) 46:56–67. doi: 10.1016/j.biocel.2013.11.011 24275097

[46] Xu E Xia X Jiang C Li Z Yang Z Zheng C. GPER1 silencing suppresses the proliferation, migration, and invasion of gastric cancer cells by inhibiting PI3K/AKT-mediated EMT. Front Cell Dev Biol. (2020) 8:591239. doi: 10.3389/fcell.2020.591239 33425895 PMC7793665

[47] Sarvari M Kallo I Hrabovszky E Solymosi N Liposits Z. Ovariectomy alters gene expression of the hippocampal formation in middle-aged rats. Endocrinology. (2017) 158:69–83. doi: 10.1210/en.2017-00514 27805868

[48] Nishida A Furukawa A Koike C Tano Y Aizawa S Matsuo I. Otx2 homeobox gene controls retinal photoreceptor cell fate and pineal gland development. Nat Neurosci. (2003) 6:1255–63. doi: 10.1038/nn1155 14625556

[49] Clark BS Stein-O’Brien GL Shiau F Cannon GH Davis-Marcisak E Sherman T. Single-cell RNA-seq analysis of retinal development identifies NFI factors as regulating mitotic exit and late-born cell specification. Neuron. (2019) 102:1111–26.e5. doi: 10.1016/j.neuron.2019.04.010 31128945 PMC6768831

[50] Molina L Bustamante FA Bhoola KD Figueroa CD Ehrenfeld P. Possible role of phytoestrogens in breast cancer via GPER-1/GPR30 signaling. Clin Sci (Lond). (2018) 132:2583–98. doi: 10.1042/CS20180885 30545896

[51] Soltysik K Czekaj P. Membrane estrogen receptors - is it an alternative way of estrogen action? J Physiol Pharmacol. (2013) 64:129–42. 23756388

[52] Conroy LR Hawkinson TR Young LEA Gentry MS Sun RC. Emerging roles of N-linked glycosylation in brain physiology and disorders. Trends Endocrinol Metab. (2021) 32:980–93. doi: 10.1016/j.tem.2021.09.006 PMC8589112 34756776

[53] Bass JJ Wilkinson DJ Rankin D Phillips BE Szewczyk NJ Smith K. An overview of technical considerations for Western blotting applications to physiological research. Scand J Med Sci Sports. (2017) 27:4–25. doi: 10.1111/sms.12702 27263489 PMC5138151

[54] Pupo M Bodmer A Berto M Maggiolini M Dietrich PY Picard D. A genetic polymorphism repurposes the G-protein coupled and membrane-associated estrogen receptor GPER to a transcription factor-like molecule promoting paracrine signaling between stroma and breast carcinoma cells. Oncotarget. (2017) 8:46728–44. doi: 10.18632/oncotarget.v8i29 PMC5564519 28596490

