# Supplementary figures and images for: G protein-coupled estrogen receptor expression in postnatal developing mouse retina
Source: Front Ophthalmol (Lausanne). 2024 Mar 15;4:1331298. doi: 10.3389/fopht.2024.1331298 (PMC11182193; doi:10.3389/fopht.2024.1331298)

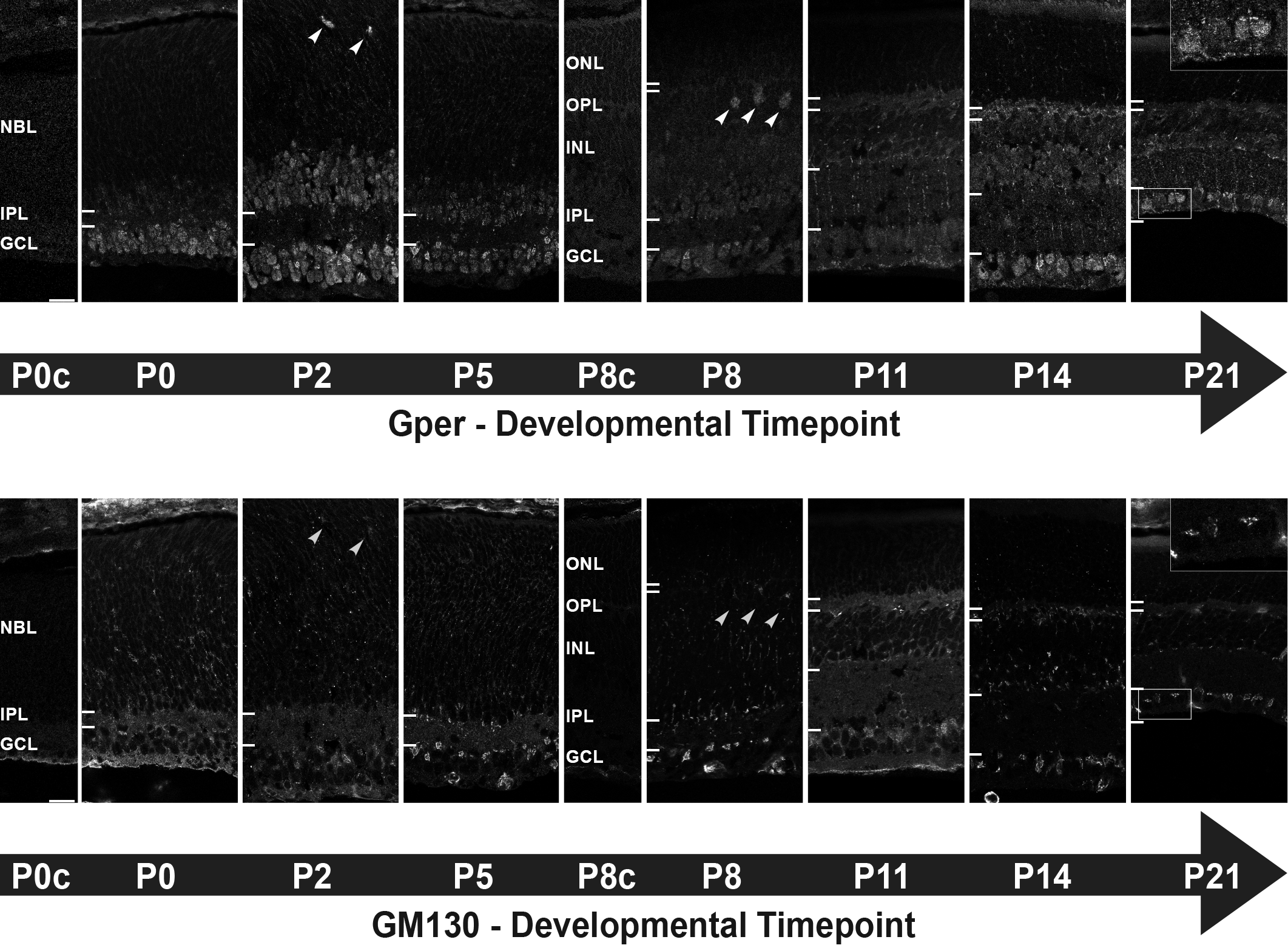

Supplement: Supplementary Figure — Gper (top) and GM130 (bottom) localization during postnatal retinal development. See legend for Figure 1F for details. [file Image_1.tif]
